# Addressing social issues in a universal HIV test and treat intervention trial (ANRS 12249 TasP) in South Africa: methods for appraisal

**DOI:** 10.1186/s12889-015-1344-y

**Published:** 2015-03-01

**Authors:** Joanna Orne-Gliemann, Joseph Larmarange, Sylvie Boyer, Collins Iwuji, Nuala McGrath, Till Bärnighausen, Thembelile Zuma, Rosemary Dray-Spira, Bruno Spire, Tamsen Rochat, France Lert, John Imrie, for the ANRS 12249 TasP Group

**Affiliations:** INSERM/University of Bordeaux, ISPED, Centre Inserm U897- Épidemiologie-Biostatistique, Bordeaux, France; Centre Population et Développement (CEPED UMR 196 Université Paris Descartes Ined IRD), Paris, France; Africa Centre for Health and Population Studies, University of KwaZulu-Natal, Durban, South Africa; INSERM-IRD-Aix-Marseille University, Faculty of Medicine, Aix-Marseille School of Economics (AMSE), SESSTIM-UMR 912, 13006 Marseille, France; Academic Unit of Primary Care and Population Sciences, and Department of Social statistics and Demography, University of Southampton, Southampton, UK; Department of Global Health and Population, Harvard School of Public Health, Boston, Massachusetts USA; INSERM, UMR_S1136, Pierre Louis Institute of Epidemiology and Public Health, Research Team in social epidemiology, F-75013 Paris, France; Sorbonne Universités, UPMC Univ Paris 06, UMR_S1136, Pierre Louis Institute of Epidemiology and Public Health, Team Research in social epidemiology, F-75013 Paris, France; Centre de recherche en Épidémiologie et Santé des Populations (CESP, Inserm Unité 1018), Villejuif, France; Centre for Sexual Health and HIV Research, Research Department of Infection and Population Health, Faculty of Population Health Sciences, University College London, London, UK

**Keywords:** HIV infections, HIV testing, Community, HIV care, Antiretroviral treatment, Social, Behaviour, Cost, South Africa

## Abstract

**Background:**

The Universal HIV Test and Treat (UTT) strategy represents a challenge for science, but is also a challenge for individuals and societies. Are repeated offers of provider-initiated HIV testing and immediate antiretroviral therapy (ART) socially-acceptable and can these become normalized over time? Can UTT be implemented without potentially adding to individual and community stigma, or threatening individual rights? What are the social, cultural and economic implications of UTT for households and communities? And can UTT be implemented within capacity constraints and other threats to the overall provision of HIV services? The answers to these research questions will be critical for routine implementation of UTT strategies.

**Methods/design:**

A social science research programme is nested within the ANRS 12249 Treatment-as-Prevention (TasP) cluster-randomised trial in rural South Africa. The programme aims to inform understanding of the (i) social, economic and environmental factors affecting uptake of services at each step of the continuum of HIV prevention, treatment and care and (ii) the causal impacts of the TasP intervention package on social and economic factors at the individual, household, community and health system level. We describe a multidisciplinary, multi-level, mixed-method research protocol that includes individual, household, community and clinic surveys, and combines quantitative and qualitative methods.

**Discussion:**

The UTT strategy is changing the overall approach to HIV prevention, treatment and care, and substantial social consequences may be anticipated, such as changes in social representations of HIV transmission, prevention, HIV testing and ART use, as well as changes in individual perceptions and behaviours in terms of uptake and frequency of HIV testing and ART initiation at high CD4. Triangulation of social science studies within the ANRS 12249 TasP trial will provide comprehensive insights into the acceptability and feasibility of the TasP intervention package at individual, community, patient and health system level, to complement the trial’s clinical and epidemiological outcomes. It will also increase understanding of the causal impacts of UTT on social and economic outcomes, which will be critical for the long-term sustainability and routine UTT implementation.

**Trial registration:**

Clinicaltrials.gov: NCT01509508; South African Trial Register: DOH-27-0512-3974.

**Electronic supplementary material:**

The online version of this article (doi:10.1186/s12889-015-1344-y) contains supplementary material, which is available to authorized users.

## Background

Four large-scale cluster randomized trials are ongoing in Eastern and Southern Africa to measure the efficacy of a Universal Test and Treat (UTT) approach in ‘real life’ [1]: ANRS 12249 TasP (Treatment–as-Prevention) in South Africa [2,3]; HPTN 071 PopART in South Africa and Zambia [4,5]; SEARCH in Kenya and Uganda [6] and the Botswana Combination Prevention project [7]. All four trials rely on some form of longitudinal population-based HIV surveillance approach to evaluate changes in HIV prevalence and, most importantly, in HIV incidence over time.

Following Granich and colleagues’ model published in the Lancet in 2009 [8], UTT interventions are built around two main components:

1. HIV counselling and testing of all, or nearly all, members of a defined population in a geographical area to identify those already infected with HIV or diagnosed but not yet linked to care, and thereafter regular and repeat HIV testing of those who test HIV-negative to identify new positives as early as possible after seroconversion;
2. initiation of life-long antiretroviral treatment (ART) as soon as possible after HIV diagnosis, regardless of CD4 count, while supporting other preventive behaviours (e.g. consistent condom-use with all partners) to further enhance the expected benefits of immediate ART.

Reports modelling the effects of UTT [9,10] suggest that the significant prevention benefits (i.e. statistically significant reductions in HIV incidence) necessary to warrant taking UTT interventions to scale are obtained only when very high levels of uptake of the two key components of the UTT strategy are achieved and sustained – as high as 90% of HIV-negative people tested for HIV every year and 90% of HIV-infected people starting ART [8]. Yet data published so far, highlight the challenge of reaching such high uptake rates of HIV testing and HIV care, even before considering a UTT strategy itself. A recent meta-analysis of home-based voluntary HIV testing in sub-Saharan Africa showed that the proportion of people who accept home-based HIV testing ranged from 58.1% to 99.8% overall (pooled percentage 83.3%), and from 58.1 to 91.8% in South Africa specifically [11]. This review did not present data on repeat HIV testing, however the few available reports on uptake of consecutive HIV testing campaigns show a 75-80% uptake of a second test among those tested the first time [12,13]. In terms of linkage to care and ART initiation, a recent meta-analysis of sub-Saharan African data published between 2001 and 2012, showed that, for 100 patients with a positive HIV test, 72 had a CD4 count performed, 40 were eligible for ART and only 25 started [14]. The 2013 World Health Organisation (WHO) guidelines on ART eligibility recommend initiation of ART at a CD4 count threshold of 500 cells/mm^3^ [15], but these new recommendations are not yet implemented by most African countries [16]. Thus there is little, if any, data on the acceptability and uptake of early or immediate ART (i.e. CD4 > 350 cells/mm^3^). In this context, and with UTT not being a single intervention of HIV testing or initiation of ART, but rather a complex combination, considerable barriers to the implementation and uptake throughout the UTT cascade can be anticipated [17,18].

As much as UTT represents a challenge for scientists, public health authorities and health care providers, it is also likely to be a challenge for individuals and societies. There is limited research addressing how communities respond to participating in a research programme that involves substantial normative social change in community cultures and perceptions. South Africans have been exposed to rapidly changing discourses from public authorities with regard to the cause of HIV/AIDS, the ways to prevent HIV infection and to care for people with HIV. UTT as a new approach to deal with the epidemic in the community might raise distrust from the community or, conversely, strong support. Preliminary qualitative enquiry [19,20] and discussion with community leaders and key informants in South Africa suggest that the UTT approach is welcome, but what the individual and community response to the interventions will be is largely unknown.

We constructed a multi-disciplinary research programme implemented as part of the ANRS 12249 Treatment-as-Prevention (TasP) trial in South Africa. In this paper, we first briefly present the overall trial design. We then outline the research questions and objectives that each component of our multi-disciplinary research programme aims to address. We describe in detail the research methods and specific data collection tools being implemented. Finally we discuss some of the emerging issues raised by UTT strategies that are unlikely to be answered in the short-term by any of the ongoing trials.

## Methods/design

### The ANRS 12249 TasP trial

The protocol of the TasP trial, registered on clinicaltrials.gov (NCT01509508), has been described elsewhere [2]. In summary, the main hypothesis of the TasP trial is that HIV testing of all adult members of a community, followed by immediate ART initiation of all, or nearly all, HIV-infected participants regardless of immunological or clinical staging, will prevent onward transmission and reduce HIV incidence in this population. The TasP trial is a cluster-randomised trial implemented in the Hlabisa sub-district, in rural northern KwaZulu-Natal in South Africa, an area with approximately 228 000 Zulu-speaking inhabitants. The HIV prevalence in the sub-district is one of the highest in the world, with around 29% of adults infected with HIV [21].

The UTT strategy being tested in the cluster-randomised TasP trial has two main components (the trial intervention package): universal and repeat home-based HIV testing of all resident adults and immediate ART initiation. In both trial arms, rounds of home-based HIV testing are repeated every six months. All trial participants identified as HIV-infected are referred to a local TasP trial clinic situated in the trial cluster in which they live. In the control clusters, HIV infected adults are offered ART according to current South African guidelines (i.e. at less than 350 CD4 cells/mm^3^ or WHO stage 3 or 4 or pregnancy). In the intervention clusters, all HIV infected adults seen in TasP trial clinics are offered the opportunity to begin ART immediately regardless of CD4 count or clinical staging.

Implementation of the trial followed a two-phased approach. The first phase started with four clusters in March 2012, six additional clusters started in January 2013, with the first round completed in all 10 clusters in March 2014 [22]. In phase 2, started in June 2014, the trial continues for two further years (4 rounds) in the 10 clusters from the first phase and extended to 12 new clusters. In total, the trial is implemented in 22 (2 × 11) clusters, expected to contribute 58 cluster-years of follow-up with an average cluster size of 1,000 residents 16 years or older, of whom an estimated 200 are living with HIV.

### Research questions addressing social issues within the TasP trial

Implementation of the trial intervention package in the Hlabisa sub-district will modify the HIV prevention and care landscape, with a likely increased number of people aware of their HIV status and with more HIV-infected people having the opportunity to initiate ART immediately after HIV diagnosis.

The social science research programme embedded in the ANRS 12249 TasP trial aims to comprehensively inform understanding of the (i) social, economic and environmental factors associated with uptake of each component of the trial intervention package (the HIV treatment and care “cascade” [23]); (ii) overall journey of trial participants through the continuum of HIV prevention and care; and (iii) social and economic impact of the trial intervention package at individual, household, community, population and health system level. These dimensions are intrinsically linked together, for example, changes in social norms are likely to have an impact on HIV testing uptake and linkage to care, and at the same time, may change as HIV testing and care practices evolve [24].

Table 1 summarizes the research questions addressed by the social research programme, grouped according to the following main topic areas: community perceptions and experiences, HIV testing, linkage to HIV care, HIV care and treatment, sexual behaviours and HIV risk and prevention practices, and economic impacts and economic value.

**Table 1:** **Summary of research questions addressed within the ANRS 12249 TasP trial social research programme and triangulation of associated surveys and sub-studies**

**Key research areas**	**Research questions**	**Home-based survey**	**Clinic-based survey**	**Community qualitative study**	**Costs assessment and time-motion survey**	**Health care professionals survey**
Community perceptions and experiences	What are the community perceptions and experiences of the trial intervention package?			X		
	How does the trial intervention package of HIV testing and care fit with community’s experience of Department of Health service provision?			X		
	Can communities be successfully engaged in the trial intervention package, i.e. does community stigma towards PLWHIV decrease and social support improve over the duration of the trial?	X	X	X		
HIV testing	What are the individuals and community attitudes to and perceptions of HIV testing and repeat HIV testing, and do these change over the duration of the trial?	X		X		
	What are the social, economic and environmental barriers to initial and repeat home-based HIV testing, and do these change over duration of the trial?	X		X		
	What is the impact of repeat HIV testing on disclosure and conjugal relationships and do these change over the duration of the trial?					
Linkage to HIV care	What are the social, economic and environmental barriers to entry into care and do these change over duration of the trial?	X	X	X		
	How acceptable to individuals and the community is the trial model of HIV care?		X	X		
HIV care and treatment	What are community and individual expectations, perceptions and knowledge of immediate ART over time?	X	X	X		
	What are the social, economic and environmental barriers to immediate ART and do these change over the duration of the trial?		X	X		
	What is the impact of immediate ART on adherence and retention in care and how does it changes over time?		X	X		
	What are the causal impacts of immediate ART for quality of life and patient satisfaction?		X			
	What are the psycho-social impacts (disclosure status, union-break up, social support, perceived stigma, depression, gender-based violence) of immediate ART?		X	X		
	What are community and individual expectations, perceptions and knowledge of immediate ART and do these change over time?	X	X	X		
Sexual behaviours and HIV risk/prevention practices	What are the most common HIV sexual risk behaviours and practices (e.g. multiple concurrent partners)?	X	X			
	What are the main prevention strategies (change in sexual practices, condom use, male circumcision) and do these changes over the duration of the trial?	X	X			
	What are the effects of immediate ART on sexual behaviours and HIV prevention practices?	X	X			
Economic impacts and economic value	What is the causal impact of the trial intervention package on employment, household welfare and private health care spending?	X	X			
	How does the trial intervention affect quality of care and health systems outcomes, such as impacts on health care professionals (training, working conditions, practices, perceptions) and health care capacity?					X
	What are the cost and the cost-effectiveness of the trial intervention package (home-based testing, immediate ART) in this rural South African context? What is the full social net value of the trial intervention package?	X	X		X	X
	What is the feasibility and financial sustainability (budget impact) of the trial intervention package (health system level) in this rural South African context?	X	X		X	X

ART: antiretroviral therapy; DoH: department of health; PLWHIV: people living with HIV.

#### Community perceptions and experiences

Implementation of immediate ART with the aim of bringing about community prevention benefits is a new concept, different from ART initiation for the sole individual clinical benefit. Treatment-as-prevention or “treatment is prevention” may change the social representations of ART, HIV transmission risk and HIV infection itself and in turn, could lead to change in HIV sexual risk and HIV prevention practice. Such a large-scale and intensive intervention has the potential to increase stigma and marginalization of HIV-infected people, or may, on the other hand, contribute to normalization of HIV and greater acceptability of people living with HIV, with increased social support. Could universal HIV testing in the trial community induce a form of “required” HIV status disclosure to partners, family and other community members? For those who do not want to disclose their HIV status, how feasible will it be to keep this knowledge confidential in a context where everyone or almost everyone will have been tested? Depending on these effects, repeat HIV testing uptake, as well as linkage to care and treatment, acceptability for people diagnosed HIV positive during home-based testing may differ. It is crucial to understand community perceptions of UTT and identify social norms that may change and affect the acceptability of the trial intervention package [25].

#### HIV testing

A UTT strategy by its nature raises questions about perceptions and practices of HIV testing in the study population. Home-based HIV testing has been shown to be an acceptable intervention in KwaZulu-Natal [26] but acceptability and feasibility of providing repeated home-based testing, as frequently as twice a year, has not been assessed. How effective is home-based HIV testing programme in reaching the entire resident population of a community? Does the acceptability of home-based HIV testing differ according to previous exposure to/experience of HIV (testing or care), personally, within the family or within the immediate surroundings? Does home-based HIV testing complement or supplement the current HIV testing provision in the trial area? Who systematically refuses home-based HIV testing? Do repeated campaigns of home-based HIV testing allow for the early identification of individuals recently infected by HIV? Finally, what impact, if any, will the rounds of repeat testing and the resulting higher levels of HIV status knowledge have on couples and their personal and sexual relationships, in particular in terms of HIV status disclosure and partnership dissolution?

#### Linkage to HIV care

Linkage to HIV care is still a major challenge for most ART programmes in sub-Saharan Africa [27]. But to ensure maximum reduction of HIV incidence, a UTT strategy requires that HIV-infected individuals are linked to care as soon as possible after seroconversion. How effective is early linkage to care in the context of repeated rounds of home-based HIV testing and what are the social, economic and environmental barriers? How do community members perceive TasP trial clinics dedicated only to HIV-infected individuals? Does the perspective of immediate ART initiation following HIV diagnosis (vs. according to current guidelines) alleviate/reinforce the various barriers to entry in care?

#### HIV care and treatment

There is growing evidence that early treatment in HIV positive individuals is associated with improved clinical benefits compared with delayed treatment [28,29]. Besides clinical benefits, immediate ART is likely to also affect psychosocial and behavioural outcomes such as adherence to ART, quality of life and retention in care, but the direction and magnitude of these effects are unknown. Will adherence be lower for people who do not experience the need for treatment as they are still in good health when initiating ART? Will quality of life decrease if individuals experience side-effects or if the treatment is not well accepted? Or will universal treatment generate a higher acceptability of HIV infected people and increased awareness of the benefits of treatment, including its prevention benefits, thus improving adherence to treatment and quality of life of HIV positive individuals initiating ART immediately? It is also plausible that there is a gain in quality of life due to UTT, at least among some patients, because they can utilize ART as soon as they want to, rather than being told that they will have to wait until they suffer from more advanced HIV disease.

#### Sexual behaviours and HIV risk/prevention practices

The nature and magnitude of the social effects of UTT are unknown and difficult to predict, especially on sexual behaviours, HIV risk and prevention practices. A key question is whether the overall effect of immediate ART on reducing HIV incidence at the community level is counterbalanced by potential disinhibition effects on sexual behaviours? Could the knowledge of the preventive effect of ART induce decreased condom use? Recent work in the same area found no evidence of increased sexual risk-taking at the population level following ART availability and even protective changes in some behaviours [30]. Emerging literature based on clinical trials assessing early ART (at CD4 > 350) also suggest that condom use is comparable among HIV-infected people treated below and above the 350 CD4 threshold, and rates of partnership acquisition and dissolution are also similar [31,32]. However, such effects need to be confirmed when early ART is scaled up at the population level and used for long periods of time.

#### Economic impacts and economic value

ART can lead to changes in employment and household welfare [33] raising the issue of the economic impact of the trial intervention package at household and community levels. Will expanding access to immediate ART induce extra expenses for individuals and households such as transportation cost, food, children’s supervision, work days lost, for example? Will immediate ART avert the economic losses that people experience before they initiate ART during comparable late disease stages [33] or will it lead to economic productivity losses because of ART side effects and time lost due to health care utilization among patients who are not yet experiencing any severe symptoms of HIV disease [34]? Or conversely, will it improve economic and social outcomes because the health losses due to advanced HIV disease are prevented rather than treated?

UTT also raises important issues about the feasibility and financial sustainability of such a public health strategy given existing resources. These include the cost of the trial intervention package and its cost-effectiveness, as well as the resource needs and budgetary impact over the long term. Difficult working conditions, inadequate training and lack of career development have been shown to have an impact on human performance and may jeopardize quality of care, as well as HIV treatment delivery [35]. To what extent do the human resources constraints challenge the implementation of a UTT strategy in the trial area?

### Research methods

**Figure 1:**
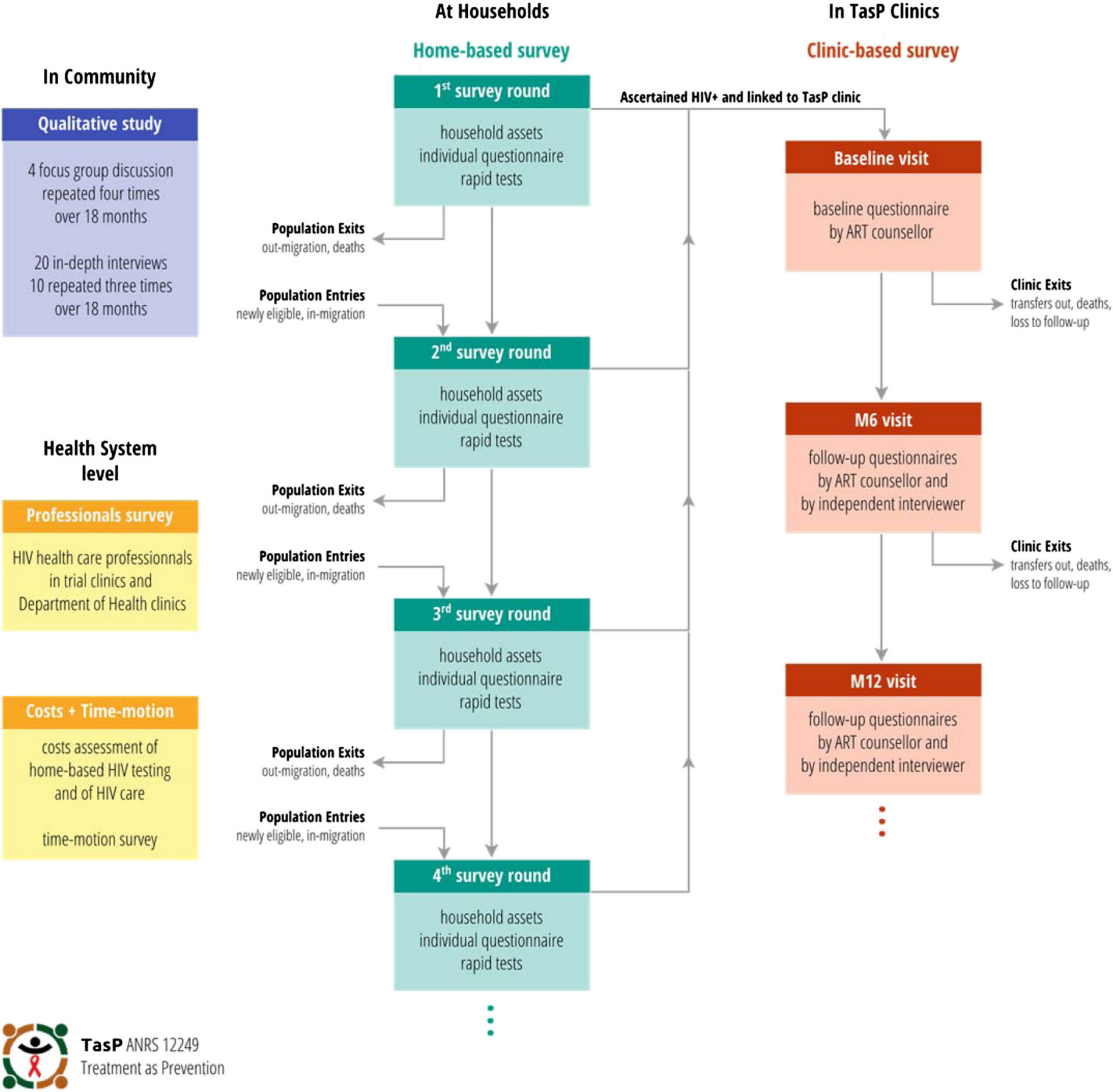
**Components of the ANRS 12249 TasP trial social research programme: surveys, populations and tools.**

To address the many social research questions raised by the trial intervention package implemented within the ANRS 12249 TasP trial, we adopted a multidisciplinary, multi-level and mixed-methods research approach. We designed several surveys, at the community, household, individual, and clinic level, combining quantitative and qualitative methods (Figure 1). Survey results will be triangulated so as to provide a comprehensive response to each of the questions outlined in Table 1.

#### Home-based survey

The home-based survey is repeated at each round of home-based HIV testing. It consists of a series of face-to-face questionnaires administered by fieldworkers/HIV counsellors: two questionnaires administered at the household level to the head of household (the household registration questionnaire and the household assets questionnaire) (see Table 2) and one questionnaire administered at the individual level to each household member who is eligible and willing to participate in the trial (see Table 3). These household and individual data will contribute to profiling the individuals and population groups who are not reached by the trial intervention package or who decline part or all of it.

**Table 2:** **Items documented in the household questionnaires**

**Home-based household questionnaire**	**Registration visit**	**Follow-up visits**
Household composition and basic socio-demographic (gender and age) characteristics	X	
Changes in household composition (including in-out migrations/mortality/newly eligible)		X
Household assets	X	X
Food security	X	X

**Table 3:** Items documented in the individual questionnaires (IQ)*

	**Phase 1 1** ^**st**^ **contact IQ1**	**Phase 1 2** ^**nd**^ contact IQ2**	**Phase 1 3** ^**rd**^ **contact IQ3**	**Phase 2 IQ**
**Home-based individual questionnaire**				
Education	X		X	X
Employment and income**	X	X	X	X
Marital status	X	X	X	X
Parenthood	X		X	X
Attitudes and beliefs about HIV infection and treatment*	X	X	X	X
HIV testing behaviour	X		X	X
Attitudes and beliefs about HIV testing	X		X	X
Knowing someone with HIV infection	X		X	X
Self-reported knowledge of HIV status	X	X	X	X
Partnerships and sexual network patterns	X		X	X
Prevention and risk behaviours:				
- Alcohol	X	X	X	X
- Condom use	X		X	X
- Male circumcision				X
Quality of life	X		X	X
Stigma towards PLWHIV	X		X	X
Health care use and expenditure		X		X
Safety and security		X		X
**Home-based HIV testing**				
Dried Blood Spot	X	X	X	X
Home HIV counselling and rapid testing	X	X	X	X

*The individual questionnaire is administered at each home-based testing rounds, i.e. theoretically every six months. Phase 1 took place between March 2012 and May 2014. Phase 2 started in June 2014.

**Questions in IQ1/IQ3 and IQ2 are slightly different. All of them are incorporated in the phase 2 IQ. The IQ2 module is a shorter version of the IQ1/IQ3 module.

PLWHIV: people living with HIV.

The core of the home-based individual questionnaire is comprised of items that have been used in previous Africa Centre research studies, particularly work conducted in the Africa Centre Demographic Surveillance Area (e.g. socio-demographics [36], sexual behaviour and sexual relationship [30], health care expenditure [37], acceptance of HIV counselling and testing and/or Dried Blood Spot [36]). In addition, to assess quality of life, we use the EQ-5D scale [38], a short five-item questionnaire validated in the isiZulu language by the EuroQol group [39].

#### Clinic-based survey

The clinical follow-up of patients enrolled in TasP trial clinics has been described in detail elsewhere [2]. We describe here the clinic-based survey implemented in each of the TasP trial clinics. All HIV-positive adults who choose to be followed in these TasP trial clinics are eligible to participate in the survey, regardless of their eligibility for ART. Trial participants who choose to remain in care in the Department of Health (DoH) clinics are not included in this survey for practical reasons.

The clinic-based survey is composed of several questionnaires, some administered by the TasP trial clinic’s ART counsellor and others by an independent interviewer. During the first clinic registration visit, the ART counsellor administers a baseline questionnaire, designed as an additional module to the baseline information collected by the Department of Health (DoH case report form). HIV-infected patients are then invited to participate in six-monthly follow-up questionnaires, administered by an independent interviewer, in order to limit social desirability bias; the ART counsellor also administers a short questionnaire for a small number of non-sensitive questions such as economic situation (see Table 4).

**Table 4:** **Items documented in the clinic-based questionnaires**

**Topic**	**M0**	**M6**	**M12**	**M18**	**M24**	**M30**	**M36**	**M42**	**M48**
ART perception	C								
ART knowledge		I	I	I	I	I	I	I	I
Self-reported adherence*^,^		I	I	I	I	I	I	I	I
Disclosure and couple union	C	I	I	I	I	I	I	I	I
Sexual behaviour		I	I	I	I	I	I	I	I
Gender attitudes and violence		I	I	I	I	I	I	I	I
Social and community support	C	I	I	I	I	I	I	I	I
Alcohol consumption	C	C	C	C	C	C	C	C	C
Depression and anxiety	C	I	I	I	I	I	I	I	I
Stigma and discrimination		I	I	I	I	I	I	I	I
HIV Quality of life		I	I	I	I	I	I	I	I
Economic situation: income, consumption and wealth	C	C	C	C	C	C	C	C	C
Health expenditure	C	I	I	I	I	I	I	I	I
Time and costs associated with the clinic visit	C	C	C	C	C	C	C	C	C
Satisfaction with care		I	I	I	I	I	I	I	I

C: ART counsellor-administered questionnaire; I: interviewer-administered questionnaire; *for participants on ART only.

A large part of the clinic-based questionnaires is comprised of items used elsewhere in this trial (e.g. sexual behaviour and sexual relationship or health care expenditure questions included in the individual home-based HIV testing questionnaire) or in previous Africa Centre research studies (e.g. satisfaction with care [40] or social support [41,42].

Internationally recognized tools and validated measurement scales are used to assess violence, adherence, depression, stigma, and quality of life.

- Violence and gender attitudes are documented using an extract of the WHO Multi-country Study on Women’s Health and Domestic Violence against Women [43].
- In addition to the assessment of adherence during the clinic-follow-up based on visual analogue scale, pill identification test and pill count [44], we assess self-reported adherence using a scale, validated in another African country context, designed to limit both recall and social desirability bias [45-48]. This tool includes several questions related to dose taking during the previous four days and adherence to the dosing time schedule during the previous four weeks. Adherence scores, which are computed using a validated algorithm allow classification of patients into highly adherent, moderately adherent and poorly adherent which has been shown to be significantly associated with viral load [46]. Another item focusing recording occurrence of treatment interruptions lasting more than two consecutive days during the previous four weeks, is also included as it has that has already been tested in another context African context [48] and has been found to be a predictor of resistance development in sub-Saharan setting [49].
- Stigma perceived in people living with HIV (PLWHIV) and experience of discrimination is assessed using the *HIV/AIDS stigma instrument for PLWHIV* (HASI-P) [50]. This scale has been developed to measure perceived stigma among PLWHIV in Southern African countries and its psychometric properties have been validated in different languages including isiZulu [50].
- Depression and anxiety are measured in the baseline questionnaire using the shortened version of the *Patient Health Questionnaire, the PHQ-4* [51,52] and then in the follow-up questionnaires using the *PHQ-9* [53,54]. A meta-analysis showed that PHQ-9 is acceptable in a wide range of settings, countries and populations [55] and both versions of the scale (PHQ-9 and PHQ-4) have been used in isiZulu in ongoing research by the Africa Centre.
- Quality of life is assessed using a scale specifically built for HIV infection - the *“Patient Reported Outcomes Quality Of Life, specific to HIV” (PROQOL-HIV)*. The PROQOL-HIV instrument comprises 43 items distributed throughout a comprehensive set of nine dimensions related to the quality of life of PLWHIV: general health, physical health and symptoms, treatment impact, health concerns, intimate relationship, emotional distress, body change, stigma and social relationships. Its psychometric properties have been evaluated and validated in different contexts (Australia, Brazil, Cambodia, China, France, Senegal, Thailand, USA) including sub-Saharan Africa and the instrument has been shown to be sensitive to differences in culture and gender [56].

#### Community qualitative study

The community qualitative study, implemented during the first phase of the trial in the four initial clusters, employs a combination of repeat one-on-one semi-structured interviews and repeat focus group discussions using complementary participatory methods (see Table 5).

**Table 5:** **Description of areas and issues coveed in repeat in-depth semi-structured interviews and focus group discussions**

	**Meeting 1**	**Meeting 2**	**Meeting 3**	**Meeting 4**
**In-depth interviews**	n = 20	n = 10	n = 10	
Topic	Access to health care in the community & knowledge of HIV status	Stigma induced by attending TasP trial clinics	Social support and disclosure Understanding of benefits of UTT	
Approach used	Personal experiences and representations	Personal experiences and representations	Personal experiences and representations	
**Focus group discussions**	n = 4	n = 4	n = 4	n = 4
Topic	Health care services in the community	Community and individual experiences and perceptions of UTT	Local cultures that facilitate and support regular and repeat testing and HIV status disclosure	Facilitators and barriers to HIV testing and ART uptake
Approach used	Individual and group narratives	Individual and group narratives	Individual and group narratives	Community walk

ART: antiretroviral treatment; TasP: treatment-as-prevention; UTT: universal test and treat.

##### Semi-structured interviews

A selected number of HIV positive and HIV negative participants (n = 20), comprising randomly mixed and purposefully selected groups of young and old, male and female participants are approached to participate in the semi-structured interview study. The HIV status of participants recruited from homes (n = 15) is unknown to the facilitator, unless disclosed by the participant during the interview, while individuals recruited from TasP trial clinics (n = 5) are known to be HIV infected. All participants are invited to participate in in-depth semi-structured interviews lasting 60–90 minutes. From this initial group of twenty (n = 20), ten (10) are invited to repeat the interview two more times, over 18 months. Each of the interviews has a specific purpose (see Table 5).

##### Repeat focus group discussions

Repeated focus group discussions with different community groups capture the overall impact of the UTT intervention across different sectors of the community. The focus group membership is purposively sampled by the facilitator and the Africa Centre Community Engagement officer. The members of each focus group also serve as key informants and expert advisors to help the trial team ensure that community entry, awareness and education plans are fully developed for the second phase of the trial. The four groups are comprised of: (i) mixed gender older (35 years and above); (ii) traditional healers (mixed age and gender); (iii) mixed gender youth (16–34 years old); (iv) mixed age and gender (16 years old and above). Each group meets on four separate occasions (see Table 5) with the same facilitator for a maximum of 2 hours, with specific thematic issues for each session. All sessions are audio recorded with participants’ written informed consent.

The last meeting includes a community walk which involves walking with community members through the community, observing, asking questions, and listening to things and places that are significant to community members in relation to HIV testing and treatment. In addition to the community walk, each participant is given a camera and asked to take five or more photos of things that they believe pose challenges for HIV testing and immediate ART initiation [57]. In the focus group discussion, each participant is given a chance to discuss the photos that best represent the barriers and facilitators of HIV testing and immediate ART initiation.

#### Costs assessment and time-motion survey

The cost of the intervention corresponds to the monetary value of resources used in producing the intervention. It is assessed by quantifying the different types of resources used for the implementation of the two main components of the intervention, by identifying their unit costs and finally by multiplying the quantities of each resource by its unit costs. Resources used for the implementation of the trial intervention package are obtained from the trial accounting/finance team and from activity reports for the home-based HIV testing component and from standardized clinical record forms (CRF) for the HIV care and treatment component. Unit costs are obtained mainly from the trial accounts team for both components and completed by additional external sources for care and treatment subsidised in the trial (like ART) or provided by DoH clinics (like hospitalization).

One important issue is to distinguish between resources used for the intervention production from those specifically consumed by research activities, which should be excluded from the cost calculation. The time-motion study of home-based HIV testing activities is a direct and continuous observation of tasks conducted by fieldworkers at home. This survey assesses the proportion of the fieldworkers’ workday spent on different activities (HIV testing but also research activities like DBS collection, questionnaires administration and data collection control) and to estimate salary costs dedicated specifically to research activities. Time taken to accomplish a task is recorded using a timekeeping device and reported using a standardized time sheet. The survey is implemented on randomly selected calendar days and conducted by the supervisors involved in the fieldwork supervision.

#### Health care professionals survey

A quantitative survey will be conducted in 2015 (during phase 2) among health care providers in charge of PLWHIV and working in the facilities included in the TasP trial, both the TasP trial clinics and the DoH fixed clinics. Data will be collected using a quantitative survey instrument previously used in a research programme with HIV care medical professionals in Cameroon [58]. Data collected will include information on socio-demographic characteristics of the health care professionals, training and experience in HIV care, working conditions, practices and knowledge about HIV and ART management, opinions about the UTT strategy.

In addition, data relating to the characteristics of the TasP trial clinics will be obtained through access to institutional reports, computer systems and interviews of each health centre’s managers and staff: types of health services, size of HIV clientele, number of ART-treated patients, human resources in charge of HIV care, working time devoted to the care of PLWHIV and staff compensation to estimate the cost of human resources involved in patient care.

### Ethics approval

Prior to the study implementation, initial meetings were organised with the Africa Centre Community Advisory Board (CAB) which is comprised of representatives of local traditional authorities, community members, local government and non-governmental organisation stakeholders. The CAB provided approval for the trial to take place in the local communities and is also kept informed of progress. Regularly scheduled community road shows are conducted both in control and intervention clusters to ensure continuous feedback between the investigators and the communities.

The social science programme of the ANRS 12249 TasP trial was approved by the Biomedical Research Ethics Committee (BREC) of the University of KwaZulu-Natal on 26 September 2012. The trial is being conducted with the permission of the KwaZulu-Natal DoH, South Africa (granted on 19 July 2011) and the South African Medicines Control Council (MCC) (granted on 28 June 2012).

Our consent procedures include: at home level, for each survey round, (i) verbal consent of the homestead’s owner to enter the homestead; (ii) verbal consent of the head of household to register household members and to contact them; (iii) written individual consent to complete the individual questionnaire and/or to provide a DBS; (iv) written individual consent for HIV rapid test; at TasP trial clinics level (v) individual written consent to receive HIV care and for collection of clinical and behavioural data by trial nurses and counsellors; (vi) separate written consent for each independent interviewer-administered questionnaire; and at community level (vii) written consent of each participant involved in focus groups or in-depth interviews.

All consent procedures and forms have been approved by the BREC. For participants aged 16 or 17, we collect both the consent of the participant and the consent of a parent or a guardian. The BREC is aware that some of the participants are minors and has approved the age range of participation and the specific consent procedure for minors.

## Discussion

Substantial social consequences may be expected, as a result to UTT implementation, such as changes in social representations of HIV transmission and prevention, of HIV testing, of ART use, and changes in individual perceptions and behaviours in terms of uptake of testing, frequency of testing, and ART initiation at high CD4 count, among others. The triangulation of several social science studies within the ANRS 12249 TasP trial will provide comprehensive insight, complementary to clinical and epidemiological outcomes, on the acceptability and effectiveness of a UTT intervention at individual (both HIV negative and positive), community, population and health system level.

The social science research programme we have designed within the ANRS 12249 TasP trial has a number of limitations. First, in spite of the robust community-based randomised controlled trial design within which the social research programme is structured, it may be difficult to disentangle the drivers of the social changes in the trial communities, from other research effects in communities under prolonged scrutiny.

Non-resident household members are not eligible to participate in the trial and only a limited number of their characteristics will be documented. Additional data collection targeting non-residents would be required to fully explore how in- and out-migration influences the feasibility and efficacy of UTT.

The social science research programme of the ANRS 12249 TasP trial, like the social science components in other ongoing UTT trials, will not be able to answer emerging public health and operational questions relating to operational scale-up of UTT interventions. In the next few years, UTT strategy, in one guise or another, is likely to be rolled-out in different low and middle-income country contexts. However bringing any UTT strategy to scale is going to be a complex process that will require more than trial results to guide the policy and process decisions. Reflecting back to Granich et al.’s original proposition [8] and the vast literature and commentary that have followed, nowhere have the mechanisms been defined for taking UTT beyond scientific enquiry, to scale as public health policy [59]. Ensuring that policy-makers and implementers give due consideration to the issues that surround such moves is essential. Additional research beyond the trials and evaluation studies will be required to provide evidence to guide policy makers to ensure all the complex interactions of factors are taken into consideration during implementation.

Finally, UTT relies on a programme of sustained intervention elements enacted simultaneously, which will likely need to be adapted and will likely evolve over time, although as yet this is not well defined. Would continuous provider-initiated regular and repeat HIV-testing remain acceptable? Or would alternative testing modalities (such as self-testing or mobile-testing) be needed to keep populations engaged and to identify new HIV infections as early as possible? As levels of understanding of UTT strategies in communities improve, will linking newly diagnosed patients – without any visible symptoms or perceived HIV-risk – into treatment and care become easier or more difficult? Will specific linkage to care interventions be required to encourage and support people to begin immediate ART treatment? Which models of care would be the most appropriate to UTT scale-up? Are there likely to be long-term social and behavioural consequences of large numbers of people in a given community knowing their HIV-status and starting treatment early? The follow-up period planned within our studies will not allow us to respond to all these questions, nor will our trial be able to address all the long term impacts of a sustained UTT intervention.

In spite of these limitations, the social science research programme of the ANRS 12249 TasP trial, designed as a rigorous and comprehensive package of studies, and employing different disciplinary approaches, will be instrumental in advancing understanding of barriers and facilitators of the continuum of HIV testing and care and the potential impact of UTT intervention strategies as public health interventions.

## Additional file

Additional file 1:
**Composition of the TasP Study Group.**

## Abbreviations

ANRS: Agence Nationale de Recherche sur le VIH et les hépatites virales (French national research agency on HIV and viral hepatitis)
ART: Antiretroviral treatment
BREC: Biomedical research ethics committee, University of KwaZulu-Natal
CAB: Community advisory board
CRF: Clinical record form
DBS: Dried blood spot
DoH: Department of Health
HASI-P: HIV/AIDS stigma instrument for PLWHIV
HIV: Human immunodeficiency virus
IQ: Individual questionnaire
PHQ: Patient health questionnaire
PLWHIV: People living with HIV
PROQOL-HIV: Patient reported outcomes quality of life specific to HIV
TasP: Treatment as prevention
UTT: Universal test and treat
WHO: World Health Organization
