# Supplementary material for: Addressing social issues in a universal HIV test and treat intervention trial (ANRS 12249 TasP) in South Africa: methods for appraisal
Source: BMC Public Health. 2015 Mar 1;15:209. doi: 10.1186/s12889-015-1344-y (PMC4351958; doi:10.1186/s12889-015-1344-y)
Supplement: Additional file 1: — Composition of the TasP Study Group. [file 12889_2015_1344_MOESM1_ESM.docx]

**Additional file 1: Composition of the TasP Study Group**

| **Name** | **Role** | **Affiliation** |
| --- | --- | --- |
| **Investigators** |  |  |
| François Dabis | Co-PI (France) | - Univ. Bordeaux, ISPED, Centre Inserm U897- Epidemiologie-Biostatistique, Bordeaux, France |
|  |  | - INSERM, ISPED, Centre Inserm U897- Epidemiologie-Biostatistique, Bordeaux, France |
| Marie-Louise Newell | Co-PI (United Kingdom) | - Africa Centre for Health and Population Studies, University of KwaZulu-Natal, South Africa  - Faculty of Medicine, University of Southampton, UK |
| Deenan Pillay | Co-PI (South Africa) | - Africa Centre for Health and Population Studies, University of KwaZulu-Natal, South Africa  - Faculty of Medical Sciences, University College London, UK |
| **Coordinators** |  |  |
| Collins Iwuji | Trial Coordinator and HIV Clinician (South Africa) | - Africa Centre for Health and Population Studies, University of KwaZulu-Natal, South Africa  - Research Department of Infection and Population Health, University College London, UK |
| Joanna Orne-Gliemann | Trial Coordinator (France) | - Univ. Bordeaux, ISPED, Centre Inserm U897- Epidemiologie-Biostatistique, Bordeaux, France |
|  |  | - INSERM, ISPED, Centre Inserm U897- Epidemiologie-Biostatistique, Bordeaux, France |
| **Study team** |  |  |
| Till Bärnighausen | Health economics | - Africa Centre for Health and Population Studies, University of KwaZulu-Natal, South Africa |
|  |  | - Dept of Global Health & Population, Harvard School of Public Health, Harvard Univ. Boston |
| Eric Balestre | Epidemiology and Biostatistics | - Univ. Bordeaux, ISPED, Centre Inserm U897- Epidemiologie-Biostatistique, Bordeaux, France |
|  |  | - INSERM, ISPED, Centre Inserm U897- Epidemiologie-Biostatistique, Bordeaux, France |
| Sylvie Boyer | Health economics | - INSERM, UMR912 (SESSTIM), Marseille, France |
|  |  | - Aix Marseille Université, UMR_S912, IRD, Marseille, France |
|  |  | - ORS PACA, Observatoire Régional de la Santé Provence-Alpes-Côte d’Azur, Marseille, France |
| Alexandra Calmy | Adult Medicine | - Service des maladies infectieuses, Hôpital Universitaire de Geneve, Genève. |
| Vincent Calvez | Virology | - Department of virology, Hôpital Pitié-Salpétrière, Paris, France |
| Marie-Laure Chaix | Virology | - EA 3620, Université Paris-Descartes, Laboratoire de Virologie, Hôpital Necker-Enfants Malades, AP-HP, Paris |
| Rosemary Dray-Spira | Social sciences | - INSERM U1018, CESP, Epidemiology of Occupational and Social Determinants of Health, Villejuif, France |
|  |  | - University of Versailles Saint-Quentin, UMRS 1018, Villejuif, France |
| Kamal ElFarouki | Social sciences | - INSERM U1018, CESP, Epidemiology of Occupational and Social Determinants of Health, Villejuif, France |
|  |  | - University of Versailles Saint-Quentin, UMRS 1018, Villejuif, France |
| Kenneth Freedberg | Modelling | - Massachusetts General Hospital, Harvard Medical School, Boston, MA, USA. |
| Kobus Herbst | Data management | - Africa Centre for Health and Population Studies, University of KwaZulu-Natal, South Africa |
| John Imrie | Social sciences | - Futures Group, Johannesburg, South Africa |
|  |  | - Centre for Sexual Health and HIV Research, Research Department of Infection and Population, Faculty of Population Health Sciences, University College London, London, UK |
| Sophie Karcher | Data management | - Univ. Bordeaux, ISPED, Centre Inserm U897- Epidemiologie-Biostatistique, Bordeaux, France |
|  |  | - INSERM, ISPED, Centre Inserm U897- Epidemiologie-Biostatistique, Bordeaux, France |
| Joseph Larmarange | Social sciences | - CEPED (Centre Population & Développement-UMR 196-Paris Descartes/INED/IRD), IRD (Institut de Recherche pour le Développement), Paris, France. |
|  |  | - Africa Centre for Health and Population Studies, University of KwaZulu-Natal, South Africa |
| France Lert | Social Sciences | - INSERM U1018, CESP, Epidemiology of Occupational and Social Determinants of Health, Villejuif, France |
|  |  | - University of Versailles Saint-Quentin, UMRS 1018, Villejuif, France |
| Richard Lessells | Adult medicine | - London School of Hygiene and Tropical Medicine, UK |
| Thembisa Makowa | Field operations | - Africa Centre for Health and Population Studies, University of KwaZulu-Natal, South Africa |
| Anne-Geneviève Marcelin | Virology | - Department of virology, Hôpital Pitié-Salpétrière, Paris, France |
| Laura March | Health economics | - INSERM, UMR912 (SESSTIM), Marseille, France |
|  |  | - Aix Marseille Université, UMR_S912, IRD, Marseille, France |
|  |  | - ORS PACA, Observatoire Régional de la Santé Provence-Alpes-Côte d’Azur, Marseille, France |
| Nuala McGrath | Epidemiology/Social sciences | - Academic Unit of Primary Care and Population Sciences, and Department of Social statistics and Demography, University of Southampton, United Kingdom |
| Kevi Naidu | Adult medicine | - Africa Centre for Health and Population Studies, University of KwaZulu-Natal, South Africa |
| Colin Newell | Data management | - Africa Centre for Health and Population Studies, University of KwaZulu-Natal, South Africa |
| Nonhlanhla Okesola | Nurse manager | - Africa Centre for Health and Population Studies, University of KwaZulu-Natal, South Africa |
| Tulio de Oliveira | Bioinformatics | - Africa Centre for Health and Population Studies, University of KwaZulu-Natal, South Africa |
| Melanie Plazy | Epidemiology/social sciences | - Univ. Bordeaux, ISPED, Centre Inserm U897- Epidemiologie-Biostatistique, Bordeaux, France |
|  |  | - INSERM, ISPED, Centre Inserm U897- Epidemiologie-Biostatistique, Bordeaux, France |
| Tamsen Rochat | Anthropology/psychology | - Africa Centre for Health and Population Studies, University of KwaZulu-Natal, South Africa |
| Bruno Spire | Health economics | - INSERM, UMR912 (SESSTIM), 13006, Marseille, France |
|  |  | - Aix Marseille Université, UMR_S912, IRD, Marseille, France |
|  |  | - ORS PACA, Observatoire Régional de la Santé Provence-Alpes-Côte d’Azur, Marseille, France |
| Frank Tanser | Epidemiology and Biostatistics | - Africa Centre for Health and Population Studies, University of KwaZulu-Natal, South Africa |
| Rodolphe Thiébaut | Epidemiology and Biostatistics | - Univ. Bordeaux, ISPED, Centre Inserm U897- Epidemiologie-Biostatistique, Bordeaux, France |
|  |  | - INSERM, ISPED, Centre Inserm U897- Epidemiologie-Biostatistique, Bordeaux, France |
| Johannes Viljoen | Virology | - Africa Centre for Health and Population Studies, University of KwaZulu-Natal, South Africa |
| Thembelile Zuma | Psychology/Social sciences | - Africa Centre for Health and Population Studies, University of KwaZulu-Natal, South Africa |

**Scientific advisory board**

- Chair: Bernard Hirschel (Switzerland)

- International experts: Xavier Anglaret (Ivory Coast), Hoosen Cooavdia (South Africa), Alpha Diallo (France), Bruno Giraudeau (France), Jean-Michel Molina (France), Lynn Morris (South Africa), François Venter (South Africa), Sibongile Zungu (South Africa)

- Community representatives: Eric Fleutelot (France), Eric Goemaere (South Africa), Calice Talom (Cameroon)

- Sponsor representatives (ANRS): Brigitte Bazin, Claire Rekacewicz

- Pharmaceutical company representatives: Golriz Pahlavan-Grumel (MSD), Alice Jacob (Gilead)

**Data safety and monitoring board**

- Chair: Patrick Yeni (France)

- Members: Sinead Delany-Moretlwe (South Africa), Nathan Ford (South Africa), Catherine Hankins (Netherlands), Helen Weiss (UK)
